# Impacts and Burden of Niemann pick Type-C: a patient and caregiver perspective

**DOI:** 10.1186/s13023-021-02105-8

**Published:** 2021-11-24

**Authors:** Eugen Mengel, Marc C. Patterson, Michael Chladek, Christina Guldberg, Christine í Dali, Tara Symonds, Lucy Lloyd-Price, Toni Mathieson, Joslyn Crowe, Claire Burbridge

**Affiliations:** 1 Institute of Clinical Science for LSD, SphinCS GmbH, Hochheim, Germany; 2 Mayo Clinic Children’s Center, Rochester, MN USA; 3 Clinical Outcomes Solutions, Chicago, IL USA; 4 Orphazyme A/S, Copenhagen, Denmark; 5 Clinical Outcomes Solutions, Folkestone, Kent UK; 6 Niemann-Pick UK, Washington, Tyne and Wear UK; 7 National Niemann-Pick Disease Foundation, Fort Atkinson, WI USA

**Keywords:** Niemann pick Type-C, NPC, Impacts, Illness burden, Qualitative

## Abstract

**Background:**

Niemann-Pick disease type C (NPC) is a debilitating condition that impacts patients’ and caregivers’ quality of life (QOL) and reduces the patient’s life expectancy. Since there is little qualitative research from the perspective of patients and family caregivers, this study explored the impact of NPC on patients’ and caregivers’ daily lives to understand the burden of disease.

**Results:**

A survey of caregivers for patients with NPC and adult patients with NPC (n = 49; patient age: 13 months–65 years) assessed NPC severity, importance of NPC symptoms, and how symptoms impacted patients’ and caregivers’ activities of daily living (ADLs) and health-related QOL (HRQOL). Follow-up interviews with a subset of survey participants (n = 28) explored the ranking of NPC symptom importance and impact on ADLs and HRQOL. Findings indicated that the most important manifestations of NPC were ambulation, swallowing, speech, fine motor skills, and cognition, which were those that had the most significant impact on ADLs and HRQOL. A wide range of ADLs were affected by NPC, mainly eating/drinking and the ability to perform daily tasks, including self-care, communicating, participating in school or work, and moving indoors as well as outside the home. Along with these impacts, there was an increased risk of experiencing dangerous or life-threatening situations leading to loss of patient independence and additional caregiver burden, often requiring changes in lifestyle such as giving up work. All aspects of patients’ and caregivers’ HRQOL were affected. Participants reported feelings of social isolation, loss of enjoyment in activities (patients), and feelings of sadness or worry (caregivers).

**Conclusions:**

Ambulation, swallowing, speech, fine motor skills, and cognition are important manifestations of NPC. ADLs and HRQOL were impaired in the majority of patients as well as their caregivers. The findings were independent of current age, age of onset of symptoms, and level of NPC disease-related disability; however, the impact increased at higher levels of disease disability. Knowing the impact of NPC on patients and caregivers is important for understanding the lived experience of NPC and for identifying potential areas of support.

## Background

Niemann-Pick disease type C (NPC) is an ultra-rare, progressive, neurodegenerative disease that occurs in approximately 1:100,000 live births [1]. NPC is a lysosomal storage disease caused by mutations in the *NPC1* (≈  95% of cases) or *NPC2* (≈ 5% of cases) genes [2, 3], which encode lysosomal proteins essential for intracellular transport and the metabolism of lipids [4, 5]. The mutation of these genes leads to NPC proteins often being misfolded or degrading prematurely. This process causes an accumulation of cholesterol and lipids within cells and impaired lysosomal function [5, 6]. As a result, NPC initially can impact liver, spleen, and lung function but predominantly manifests as a progressive, neurogenerative disease leading to premature death. The clinical presentation and progression of NPC is heterogeneous, varying by age at the time of symptom onset; NPC most often manifests as a loss of motor function, coordination, and speech along with cognitive impairment [7–10]. Individuals whose neurological symptoms begin in early childhood generally have a faster disease progression than patients whose symptoms begin later [7]. There is no cure for NPC. However, in recent years, several drug treatments have been considered for orphan drug status for NPC by the United States (US) Food and Drug Administration (FDA), including cyclodextrin [11, 12], miglustat [13], and most recently arimoclomol [14].

Regulatory agencies such as the FDA have increasingly emphasized the need to understand in greater detail clinical manifestations of rare diseases, their impact on individual patients and their families, and available treatments. For NPC in particular, this was highlighted during a recent patient-focused drug development (PFDD) meeting [15]. To know the true impact and burden of the condition, both the patients’ and caregivers’ perspective on NPC are important for understanding how the disease impacts their day-to-day lives given the progressive and neurodegenerative aspects of NPC, which lead to increased need for caregiving as NPC progresses. As treatments become available that may slow the progression of NPC, it is important to gain the perspectives of patients and caregivers regarding their needs and how treatments may affect these needs.

Prior research has suggested that NPC has a significant impact on patients’ and caregivers’ quality of life (QOL) [16], especially on patients’ school or work lives and caregivers’ lives as NPC progresses [17]. However, little qualitative research has been undertaken to understand more fully the impacts of NPC from the perspective of patients and caregivers. Interviews with patients and caregivers allow for a better understanding of NPC’s burden of illness on families, including its impacts on their day-to-day lives, by capturing patients’ and caregivers’ unique experiences.

Although the findings highlight the substantial burden associated with NPC for all, the precise nature of the impact and experience of NPC may differ depending on the range of symptoms experienced, the current age of the patient, and the patient’s age at symptom onset, which varies widely. Neonatal or infantile onset often leads to a more rapidly progressing fatal disorder while adult onset often manifests as a slowly progressing neurodegenerative disease [17]. Patients may also experience different sets of symptoms (e.g., neuromotor or cognitive symptoms) at different ages [18–20]. Such heterogeneity of symptoms experienced and what timeframe they manifest will have different impacts on patients’ and caregivers’ lives. While prior research has noted the clinical importance of age of symptom onset and cluster of symptoms experienced, little research has been done looking at how these aspects of NPC affect the way it impacts patients and caregivers.

The purpose of this research was to explore how NPC impacts the daily lives of patients and caregivers and to understand the burden of disease, which is the cumulative consequences of NPC, including health and social aspects of the disease. The gap between an ideal situation where one is free of disease and disability, and the cumulated health status of having NPC, is defined as the burden of disease [21]. Activities of daily living (ADLs) are an indicator of a person’s functional status that is used to collectively describe fundamental skills required to independently care for oneself such as eating, bathing, and mobility [22]. Health-related QOL (HRQOL) is a multi-dimensional concept that includes domains related to physical, mental, emotional, and social functioning. It goes beyond direct measures of population health, life expectancy, and causes of death, and focuses on the impact health status has on QOL [23]. Given the heterogeneity of symptom experience and general differences between child- and adult-onset, this research also sought to explore how the symptoms experienced lead to different impacts on patients’ and caregivers’ lives.

## Results

### Study population

A total of 49 surveys were completed across two countries: 37 in the US and 12 in the United Kingdom (UK). Of the 49 completed surveys, 43 (87.8%) were completed by caregivers (reporting for 22 [51.2%] pediatric patients and 21 [48.8%] adult patients), and six (12.2%) of the surveys were completed by adult patients reporting for themselves. A subset of 28 survey participants completed the follow-up telephone interview (20 in the US and eight in the UK). Of those interviewed, 23 (82.1%) were caregivers (reporting for 10 [43.5%] pediatric patients and 13 [56.5%] adult patients), and five (17.9%) were adult patients. Of the 43 caregivers completing the survey, 40 were a parent of the patient, one sibling, one grandparent, and one aunt. All caregivers who were interviewed were parents. Besides relation to the patient, no additional demographics about the caregivers were collected in order to focus on the patient and reduce participant burden of completing the survey. Given the low number of adult patients reporting for themselves (i.e., six in the survey and five in the interview), the impact of NPC on patients’ ADLs and HRQOL are reported primarily from the point of view of caregivers. Overall, a broad range of current ages, age of onset, and NPC severity were included in both the survey and interview (Table 1).

**Table 1:** Baseline characteristics by study activity

Baseline characteristic		Survey	Interview
Total	n	49	28^a^
Gender of patient, n (% of column total)	Female	24 (49.0)	10 (35.7)
	Male	25 (51.0)	18 (64.3)
Current age of patient, n (% of column total)	< 18 years	22 (44.9)	10 (35.7)
	18–29 years	13 (24.5)	9 (28.6)
	≥ 30 years	14 (30.6)	9 (35.7)
Reported age of onset of first NPC-related symptom, n (% of column total)^b^	< 3 months	8 (16.3)	6 (21.4)
	3 months to < 2 years	7 (14.3)	2 (7.1)
	2 to < 6 years	7 (14.3)	2 (7.1)
	6 to 15 years	14 (28.6)	10 (35.7)
	> 15 years	13 (26.5)	8 (28.6)
5-domain NPCCSS score total (0–25), n (% of column total)^c^	≤ 4	7 (14.3)	4 (14.3)
	5–9	13 (26.5)	7 (25.0)
	10–14	12 (24.5)	7 (25.0)
	15–19	11 (22.4)	7 (25.0)
	≥ 20	6 (12.2)	3 (10.7)

^a^Four of the 28 interview participants were caregivers of two individuals with NPC. However, the interviews focused on the first individual diagnosed with NPC. The characteristics reported here are of the 28 patients who were the focus of the interview

^b^Age of symptom onset based on patient self-report or caregiver report

^c^Higher scores indicate greater severity

### Importance of symptoms

In the web survey, of the nine symptom domains participants were asked to rank in order of importance (see Table 8 for a definition of each as presented in the survey), the six symptom domains most frequently ranked in the top five of importance by 40% or more of participants were: (1) Ambulation; (2) Swallow; (3) Speech; (4) Memory; (5) Cognition; and (6) Fine motor. A summary of participants’ ranking of symptom importance is in Table 2. In the interviews, participants often noted that they regarded memory and cognition as difficult to differentiate.

**Table 2:** Ranking of most important symptoms in survey

	# Times ranked n (% of row total)					# Times in top five n (% of total survey participants [49])
	First	Second	Third	Fourth	Fifth	
Ambulation	10 (24.4)	12 (29.3)	9 (22.0)	4 (9.8)	6 (14.6)	41 (83.7)
Swallow	8 (22.9)	6 (17.1)	8 (22.9)	8 (22.9)	5 (14.3)	35 (71.4)
Speech	1 (3.1)	4 (12.5)	9 (28.1)	8 (25.0)	10 (31.3)	32 (65.3)
Memory	3 (10.7)	4 (14.3)	5 (17.9)	9 (32.1)	7 (25.0)	28 (57.1)
Cognition	8 (34.8)	4 (17.4)	4 (17.4)	4 (17.4)	3 (13.0)	23 (46.3)
Fine Motor	2 (9.1)	8 (36.4)	2 (9.1)	5 (22.7)	5 (22.7)	22 (44.9)
Eye Movement	2 (10.5)	3 (15.8)	5 (26.3)	2 (10.5)	7 (36.8)	19 (38.8)
Seizures	8 (57.1)	1 (7.1)	0	3 (21.4)	2 (14.3)	14 (28.6)
Hearing	2 (18.2)	3 (27.3)	3 (27.3)	1 (9.1)	2 (18.2)	11 (22.4)

There were no major differences observed in the rankings of symptom importance when participants were grouped according to current age of the patient, nor when they were grouped according to age at symptom onset. However, there were some differences in the rankings of symptom importance when grouped by disease severity (i.e., the 5-domain NPC Clinical Severity Scale [NPCCSS] score as reported by patients or caregivers). When comparing those in the least severe to most severe group on the 5-domain NPCCSS (total score ≤ 4 versus ≥ 20), those in the least severe group more frequently ranked ambulation and cognition in their top five of importance (n = 6/7 [85.7%] vs. n = 4/6 [66.7%] and n = 5/7 [71.4%] vs. n = 2/6 [33.3%], respectively). Conversely, those in the more severe group more frequently ranked swallowing in their top five of importance than those in the less severe group (n = 6/6 [100%] vs. n = 3/7 [42.9%]).

The greater frequency of swallowing’s importance was also highlighted qualitatively in the interviews when caregivers discussed their reasons for ranking swallowing in their top five of importance. As one caregiver of a 24-year-old male with a 5-domain NPCCSS total score of 15 noted about what the increased risk swallowing difficulties meant, “I’d rather keep him from choking. […] Things that could cause him to die, those are my scary things.” Similarly, the caregiver of a 28-year-old male with a 5-domain NPCCSS score of 23 said, “He’s [at] risk feeding at the moment um, and we have recently had an appointment for uh-uh a PEG feed. […] He’s not had any chest infections yet, but it has deteriorated.”

### Overview of impacts: results of web survey

In the web survey, participants reported a variety of symptoms having an impact on the patient’s own ADLs and HRQOL as well as that of the caregiver or other family members as summarized in Table 3. Among caregivers reporting in the survey, the domains most commonly reported to impact patients ADLs and HRQOL “a lot” or “extremely” were the patient’s ambulation, swallowing, and cognitive symptoms. Caregivers reported speech and fine motor skills as impacting patients’ ADLs and HRQOL, too, but more frequently endorsed the response options of “somewhat” or “moderately” for these symptoms.

Overall, for each symptom domain, a broad range of impact was reported (from “not at all” to “extremely”), suggesting that the individual experience may be quite heterogeneous. However, the majority of participants reported being impacted by NPC to some extent (i.e., at least two-thirds reported some level of impact on each symptom). Even though patients overall were reported in the survey by their caregiver to be at a moderate disease level (mean 5-domain NPCCSS score = 12.0), 71.4% or more reported the patient’s ADLs or HRQOL being impacted at least “somewhat” by each symptom. Similarly, for the six adult patients who reported for themselves and who were at a less severe disease level (mean 5-domain NPCCSS Score = 7.8), 67.7% or more reported their own ADLs or HRQOL as being impacted at least “somewhat” by each symptom.

**Table 3:** Rating of impact of NPC symptoms on ADLs and HRQOL of patients and family/others

	Patient self-reported (n = 6)				Caregiver reported (n = 42)*			
	Impact on patients		Impact on family/others		Impact on patients		Impact on family/others	
	ADLs	HRQOL	ADLs	HRQOL	ADLs	HRQOL	ADLs	HRQOL
Ambulation, n (%)								
Not at all	1 (16.7)	2 (33.3)	2 (33.3)	2 (33.3)	5 (11.9)	6 (14.3)	6 (14.3)	10 (23.8)
Somewhat or moderately	2 (33.3)	1 (16.7)	1 (16.7)	2 (33.3)	15 (35.7)	13 (31.0)	21 (50.0)	15 (35.7)
A lot or extremely	3 (50.0)	3 (50.0)	3 (50.0)	2 (33.3)	22 (52.4)	23 (54.8)	15 (35.7)	17 (40.5)
Speech, n (%)								
Not at all	2 (33.3)	2 (33.3)	2 (33.3)	2 (33.3)	7 (16.7)	9 (21.4)	13 (31.0)	13 (31.0)
Somewhat or moderately	1 (16.7)	2 (33.3)	2 (33.3)	2 (33.3)	20 (47.6)	17 (40.5)	22 (52.4)	22 (52.4)
A lot or extremely	3 (50.0)	2 (33.3)	2 (33.3)	2 (33.3)	14 (33.3)	15 (35.7)	6 (14.3)	6 (14.3)
Swallow, n (%)								
Not at all	2 (33.3)	1 (16.7)	3 (50.0)	3 (50.0)	12 (28.6)	11 (26.2)	13 (31.0)	15 (35.7)
Somewhat or moderately	3 (50.0)	4 (66.7)	2 (33.3)	2 (33.3)	9 (21.4)	12 (28.6)	20 (47.6)	15 (35.7)
A lot or extremely	1 (16.7)	1 (16.7)	1 (16.7)	1 (16.7)	19 (45.2)	17 (40.5)	8 (19.0)	10 (23.8)
Fine motor skills, n (%)								
Not at all	1 (16.7)	1 (16.7)	2 (33.3)	2 (33.3)	7 (16.7)	9 (21.4)	11 (26.2)	14 (33.3)
Somewhat or moderately	3 (50.0)	4 (66.7)	3 (50.0)	3 (50.0)	19 (45.2)	16 (38.1)	21 (50.0)	19 (45.2)
A lot or extremely	2 (33.3)	1 (16.7)	1 (16.7)	1 (16.7)	15 (35.7)	16 (38.1)	9 (21.4)	8 (19.0)
Cognition, n (%)								
Not at all	2 (33.3)	1 (16.7)	2 (33.3)	2 (33.3)	5 (11.9)	4 (9.5)	8 (19.0)	9 (21.4)
Somewhat or moderately	4 (66.7)	5 (83.3)	4 (66.7)	4 (66.7)	13 (31.0)	14 (33.3)	17 (40.5)	19 (45.2)
A lot or extremely	0	0	0	0	22 (52.4)	23 (54.8)	15 (35.7)	12 (28.6)

*Although 42 caregivers completed the survey overall, two did not fully complete all symptoms questions; n=41 completed speech and fine motor skills items and n=40 completed swallowing and cognition items

### Key impacts on patients

**Fig. 1:**
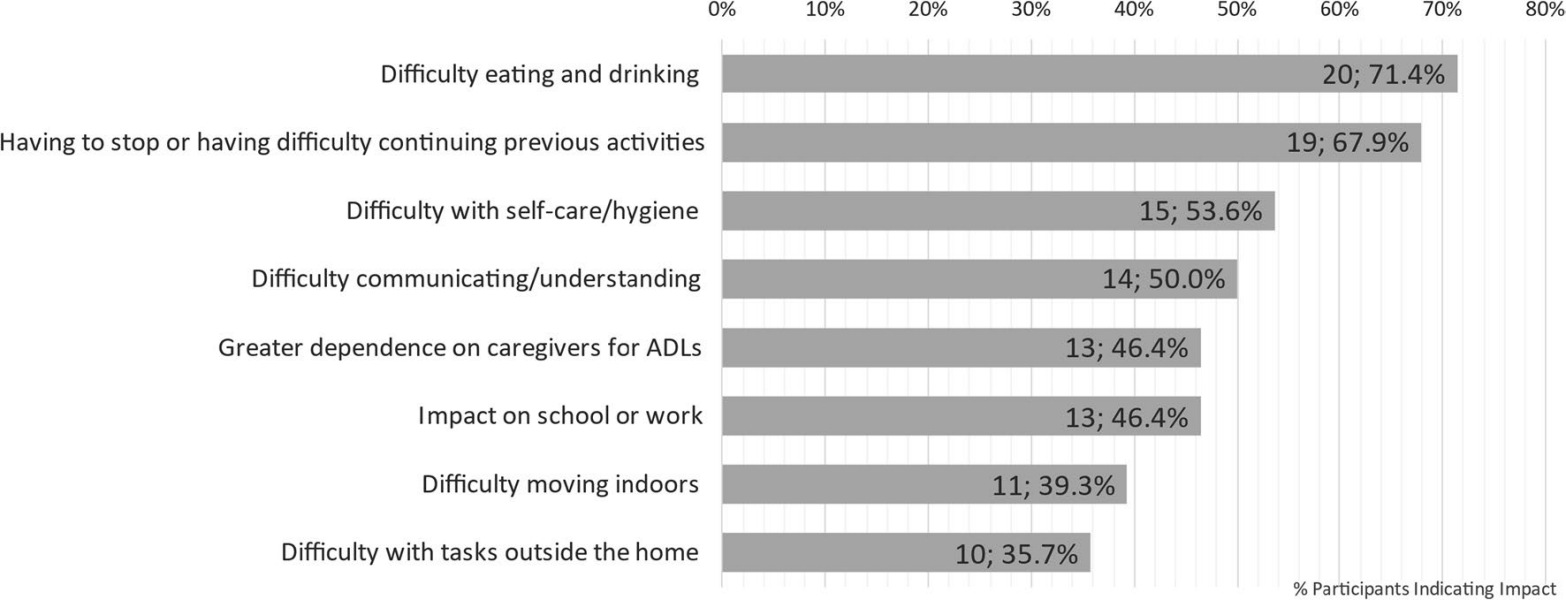
Impact of NPC on patients’ ADLs

**Fig. 2:**
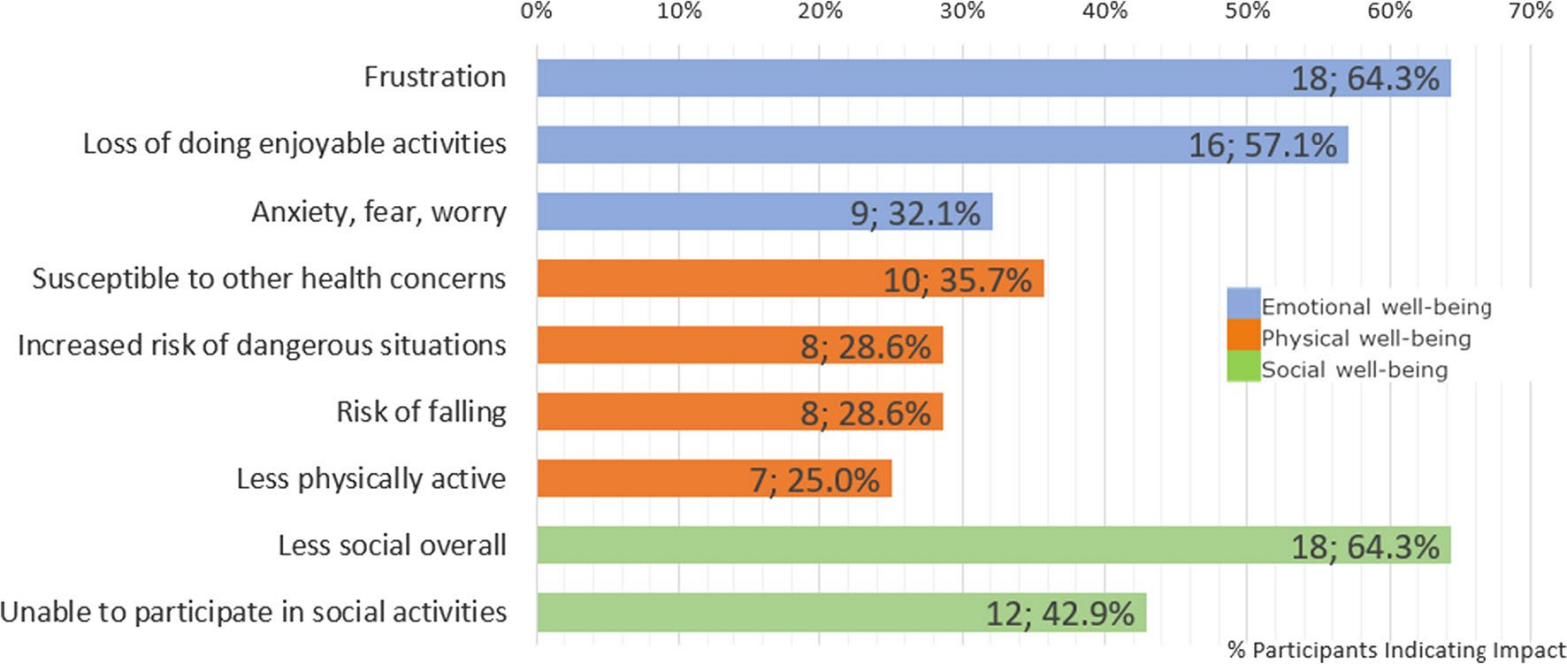
Impact of NPC on patients’ HRQOL

NPC had a considerable impact upon both ADLs and HRQOL of patients and the two, ADLs and HRQOL, were interrelated. In the interviews, caregivers and patients reported NPC symptoms had an impact on several ADLs, such as difficulties with fine motor skills and swallowing impacting patients’ eating or drinking, and having to stop or having difficulties with previous activities, which were most commonly reported, and problems with self-care and personal hygiene as summarized in Fig. 1. These impacted ADLs in turn affected patients’ HRQOL (see Fig. 2). Example quotes are given in Table 4 to illustrate the impacts of NPC on ADLs.

**Table 4:** Key impacts on ADLs of patients with example quotes

Impact on ADLs	Example Quote
Difficulty eating or drinking (n = 20; 71.4%)	“We’ve had other choking events in the past and, um, sometimes it can just even be related to if say, like, if they were raw vegetables, you know? Like, so, now we know they definitely have to be cooked and soft. Um, uh, and then the, the presentation is usually he, um, you, you know, gets real red in the face, his eyes start to bug up a little bit, and he doesn’t realize that he has to stop eating, and then he keeps shoveling more in there, and then he goes to take a drink, and then that’s when nothing will go down.” (Caregiver of 25-year-old male)
Having to give up or having difficulty continuing previous activities (n = 19; 67.9%)	“I just haven’t got the physical strength to do the things that I would normally do. I just haven’t got the energy to, it’s not every day, but most days I just haven’t got the energy to be able to do the things that I used to do.” (55-year-old male patient)
Difficulty understanding/‌communicating (n = 15, 53.6%)	“And it’s hard to, like, get her attention back on what I’m trying to convey to her, or what I’m trying to ask her or get her to do. She can’t follow, like, two step directions. Um, I could tell her, like, let’s go to the bathroom and brush your teeth, like you start walking to the bathroom and then she throws her hands up and starts singing and turns around and walks the other way, it’s like she forgot what we were even doing.” (Caregiver of 8-year-old female)
Difficulty with self-care hygiene (n = 14; 50.0%)	“You know, he just can’t control the hand there. Enough to - you know, put the toothbrush in his mouth, and like when I have to put his own deodorant on too, it takes, um, a minute or two cause, you know, his hand shake and under his armpit, or he can’t hold his other up long enough to, you know, to put the, um, deodorant on. Um, you know, as far buttoning is concerned, can’t button.” (Caregiver of 24-year-old male)
Greater dependence on caregivers for ADLs (n = 13; 46.4%)	“He has to have someone with him all the time. To h-to help him get up, to sit down, to- to do everything really whereas I- I don’t know if he remembers anyway but he, he was very independent.” (Caregiver of 28-year-old male)
Impact on school or work (n = 13; 46.4%)	“So it impacted him even at the elementary school level, um, with things like he couldn’t be a school patrol when he wanted to be. Um, and then, you know, later in middle school, he wanted to be like a part of a leadership team. And he just, the cognition isn’t there. So he, ‘cause he was a very outgoing personality but didn’t have the cognitive ability to follow through on the written portions and those kinds of things.” (Caregiver of 39-year-old male)
Difficulty moving indoors (n = 11; 39.3%)	“I walk very slowly. Um, and, so I- I, you know, when I- I’m going somewhere I do two or three things there. Like I’ll, uh, um, go the drinking fountain, go to printer, and go to the bathroom all at the same time at work. Instead of individuating them. Um, because that’s just more walking than I want to do.” (51-year-old female patient)
Difficulty with tasks outside the home (n = 10; 35.7%)	“It also impacts any kind of out of the house activities. So you know, a trip to the zoo is much more difficult at this point because of the walking that’s involved. Um, so anything out of the house becomes, um, more of a- a- a, planned kind of chore. Um, and so we kind of have to be mindful of that.” (Caregiver of 32-year-old male)

These impacts on patients’ ADLs arose from several different symptoms. Difficulty eating or drinking was often reported as a result of the patient’s swallowing difficulties (n = 17; 60.7%) or difficulties with fine motor skills (n = 12; 42.9%). Having to give up, or having difficulty continuing, previous activities often resulted from difficulties with fine motor skills (n = 12; 42.9%) and cognitive difficulties (n = 8; 28.6%). Difficulties with speech (n = 11; 39.3%) and cognition (n = 4; 14.3%) most frequently led to the impact of having difficulty understanding/communicating. Ambulation symptoms often contributed to patients’ having difficulty moving indoors (n = 7; 25.0%), having difficulty with tasks outside the home (n = 6; 21.4%), and needing assistance with daily tasks (n = 8; 28.6%), including self-care activities such as going to the bathroom (n = 4; 14.3%). Impacts on school or work often stemmed from cognitive difficulties (n = 8; 28.6%) or difficulties with memory (n = 7; 25.0%).

NPC and the impact it has upon ADLs also has a further effect upon patients’ HRQOL in a number of ways, with considerable impact on all major domains of HRQOL: emotional (n = 26; 92.9%), physical (n = 24; 85.7%), psychological (n = 10; 35.7%), and social (n = 22; 78.6%). The most frequently cited (n = 18; 64.3%) emotional impact on patients from the caregiver perspective was patients feeling frustrated about their disease progression across multiple symptoms including speech, fine motor, and cognition or memory. Regarding the physical well-being aspects of patients’ HRQOL, participants discussed issues directly related to NPC such as the risks of dangerous situations, falling and being less active, but most frequently reported patients being more susceptible to other health concerns, such as weight loss, sleep inversion, or respiratory problems (n = 10; 35.7%). In terms of social well-being, it was commonly reported that NPC made patients less social overall and more socially isolated because they found it more difficult to travel to visit others or because they had greater difficulty communicating. Example quotes are given in Table 5 to illustrate these impacts upon HRQOL.

**Table 5:** Key impacts on HRQOL of patients with example quotes

Aspect of HRQOL	Impact	Example Quote
Emotional well-being (n = 26; 92.9%)	Frustration (n = 18; 64.3%)	“I’m sure it kind of frustrates her because you know, we don’t know exactly what she needs.” (Caregiver of 19-year-old female)
	Loss of doing enjoyable activities (n = 16; 57.1%)	“He wanted to work with kids […] being a camp counselor […], but as he declined he had to leave those things as well. So the things that he found throughout his life that he wanted to do, he was unable to because of the cognition decline.” (Caregiver of 39-year-old male)
	Anxiety, fear, worry (n = 9; 32.1%)	“I’d be in an airport and I’d be looking around, although the signs, the signs would tell me to go there, I would not necessarily read the signs properly and it would register with me where I’ve actually got to go. And then that’s when most of the anxiety comes in and um, then you’d get more stressed, don’t you? And one thing sort of leads to another and the anxiety.” (55-year-old male patient)
Physical well-being (n = 24; 85.7%)	Susceptible to other health concerns (n = 10; 35.7%)	“She has been losing weight ‘cause she wasn’t able to, taking so long to feed herself, she’d give up.” (Caregiver of 18-year-old female)
	Increased risk of dangerous situations (n = 8; 28.6%)	“We’ve taken to locking the, um, the door at home now, you know, in a way that he can’t, he can’t let himself out because he would […] open the front door and go out into the garden and through the gate and, you know, he, he’s got no sense of danger or, or you know getting lost or anything like that.” (Caregiver of 8-year-old male)
	Risk of falling (n = 8; 28.6%)	“It plays so much into her ability to move around and often time, um, it results in a fall if she’s not very careful about being able to see where she’s walking or not trip over something.” (Caregiver of 19-year-old female)
	Less physically active (n = 7; 25.0%)	“We’ve noticed over the last probably year that he is more sedentary. Um, so he’ll spend quite a bit of time just sitting, um, in his recliner.” (Caregiver of 32-year-old male)
Social well-being (n = 22; 78.6%)	Less social overall (n = 18; 64.3%)	“So he was very social and then had friends and engaged in the end with this goofy little kid and he doesn’t engage with people anymore, so he doesn’t particularly have friends. Um, you know, those other kids miss class and then maybe they talked to him a little bit, but he doesn’t give much back.” (Caregiver of 16-year-old male)
	Unable to participate in social activities (n = 12; 42.9%)	“I know that she longs to be able to run and play with her friends, because I can see it in her eyes, and she can’t. So her gross motor skills are at 15 months and her fine motor skills are at 26 months, right now.” (Caregiver of 8-year-old female)

### Key impacts on caregivers

**Fig. 3:**
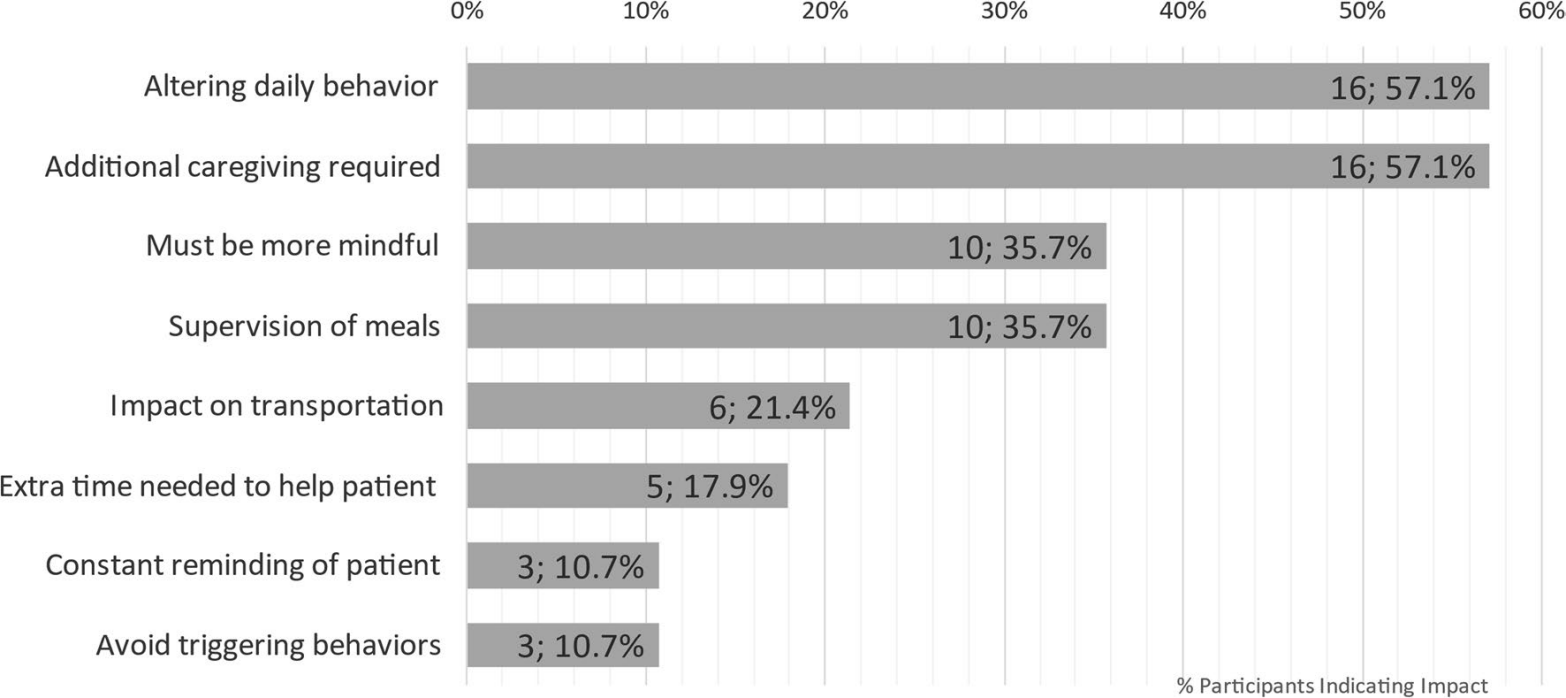
Impact of NPC on caregivers’ ADLs

**Fig. 4:**
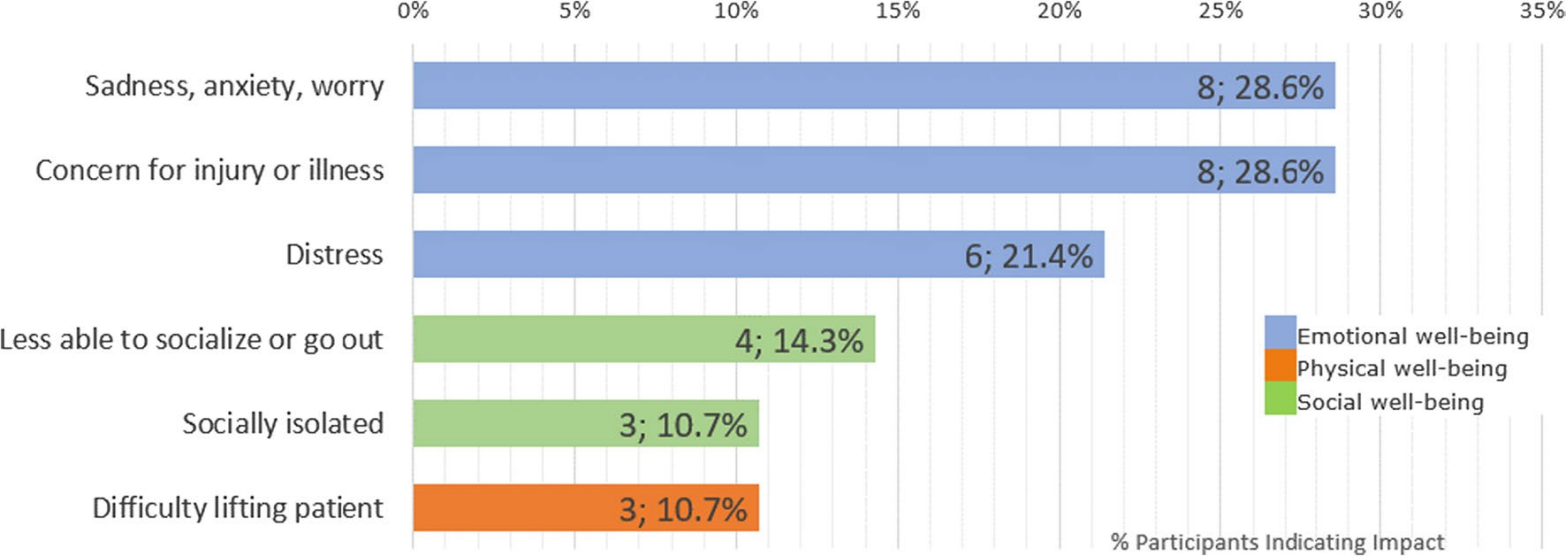
Impact of NPC on caregivers’ HRQOL

NPC not only impacted patients’ ADLs and HRQOL but also that of the caregivers (see Figs. 3 and 4). Participants frequently noted that NPC led to a change in caregivers’ daily lives, requiring changes to daily behavior and additional demands upon their time and mental energy. Practical aspects of life such as increased supervision of meals and challenges related to transportation, particularly the difficulties or adjustments needed to accommodate the patient’s wheelchair when travelling were also discussed as affecting the caregivers’ ADLs. A summary of these findings with example quotes from the interviews is presented in Table 6.

**Table 6:** Key impacts on ADLs of caregivers with example quotes

Impact on ADL	Sub-concept	Example quote
Altering daily behavior (n = 16; 57.1%	Must be more mindful (n = 10; 35.7%)	“It’s something that you just have to be mindful of and always, you know I guess on guard, and watch her. Like I usually hook my foot, like, around her chair, because if she has a seizure she can push back on her f-, with her feet, and like you know, flip the chair and go.” (Caregiver of 19-year-old female)
	Constant reminding of patient (n = 3; 10.7%)	“It impacts a lot because you know, if I forget, then she could ultimately miss something important. Like she had something, uh, yesterday, she had an appointment, and I forgot to remind her about it, forgot to do the pre-work for it, so, you know, consequently she missed it.” (Caregiver of 22-year-old female)
	Avoid triggering behaviors (n = 3; 10.7%)	“It’s a pretty cruel symptom to have [seizures] because it happens as you may or may not know, it, it happens in the context of laughter, and so what you inevitably end up doing is trying to sort of avoid making him laugh, and you know, that’s pretty horrible situation for an eight year old that, you, you know, you just, you’re a killjoy the whole time.” (Caregiver of 8-year-old male)
Additional caregiving required (n = 16; 57.1%)	Supervision of meals (n = 10; 35.7%)	“Somebody has to be with him all the time when he’s eating and kind of monitor him. Um, yeah, just, just monitoring his eating.” (Caregiver of 16-year-old male)
	Extra time needed to help patient (n = 5; 17.9%)	“Um, well it’s, like, in the morning I feel like I, you know, have to get two people ready versus me. You know, it’s a little bit harder.” (Caregiver of 19-year-old female)
Impact on transportation (n = 6; 21.4%)	N/A	“We have to make sure that we can have um, ‘cause he does use a, um, transport wheelchair when he gets really tired, as well. So, all of that has to be taken into account. So it just changes your daily life.” (Caregiver of 39-year-old male)

The impact of NPC on caregivers’ HRQOL was reported by many participants (n = 15; 53.6%) as summarized in Table 7, which also presents example quotes to illustrate the impact. Emotional impacts were those most commonly reported by caregivers, who discussed feelings of sadness, worry, anxiety, or distress about the impacts NPC was having on the patient, and reported feeling they were always on alert, being concerned about the patient suffering an injury, illness, or deteriorating due to their NPC. Caregivers also reported impacts on their own social well-being, with limitations to their activities and feelings of isolation, and physical impacts for some who had difficulty lifting the patient.

**Table 7:** Key impacts on HRQOL of caregivers with example quotes

Aspect of HRQOL	Impact	Example quote
Emotional well-being (n = 12; 42.9%)	Sadness, anxiety, worry (n = 8; 28.6%)	“Yeah, it af- affects me as well. There’s no doubt about it. Like I’ve become like I think I have kinda my doctor thinks I have PTSD from long term care-taking, you know, and I waited all those years for the bomb to drop.” (Caregiver of 24-year-old male)
	Concern for injury or illness (n = 8; 28.6%)	“It’s just scary, the safety issue because I am afraid that he will fall and, you know, break his nose or something.” (Caregiver of 2-year-old male)
	Distress (n = 6; 21.4%)	“You know that’s your kid’s future. Like how do you live with that? You don’t. I’m sorry. I’m getting emotional. It’s just, it’s like you just can’t, uh, you just, it’s just a crazy thing to, to deal with.” (Caregiver of 24-year-old male)
Social well-being (n = 6; 21.4%)	Less able to socialize or go out (n = 4; 14.3%)	“The bigger issue is, uh, you know, when we want to go out, you know, with friends or family and everything and we go out to a restaurant and he has these troubles and, you know, it’s just… you- you… it gets hard on us.” (Caregiver of 21-year-old male)
	Socially isolated (n = 3; 10.7%)	“We can’t plan to go out because i- if it happens while you’re out, what, what’s gonna happen? Who’s gonna be there?” (Caregiver of 28-year-old male)
Physical well-being (n = 3; 10.7%)	Difficulty lifting patient (n = 3; 10.7%)	“It’s physically hard on my husband and I, um, somebody’s back always hurts, is we’re always looking at each other who’s turn it is to, to help him” (Caregiver of 16-year-old male)

### Impact of NPC by degree of disability

How exactly NPC impacted patients and caregivers depended on the degree of disability. When speaking specifically about ambulation, the majority of participants reported that it impacted ADLs or HRQOL at least somewhat (Table 3). Participants across all degrees of disability discussed the impact ambulation symptoms had specifically on patients’ needing assistance with daily tasks (n = 8; 28.6%). Those with a lower degree of disability tended to talk more about problems indoors; of the seven (25.0%) participants who spoke about this, most (n = 5/7; 71.4%) had severity scores of between 5 and 9 on the 5-domain NPCCSS. However, at higher degrees of disability, ambulation symptoms appeared to have a greater impact on patients’ activities outside the home, required a greater need for assistance, or were more likely to be associated with having to stop doing certain activities. Of the six (21.4%) participants who spoke about ambulation impacting patients’ tasks outside the home, most (n = 4/6; 66.7%) had a severity score of 10 or greater.

How NPC impacted a patient’s ability to communicate or understand communication also varied depending on the degree of disability. In those at lower levels of disability (n = 11 with a 5-domain NPCCSS score of less than 10), a typical impact reported was needing to repeat oneself or having difficulty pronouncing words (n = 3/11; 27.3%), which was noted by a 51-year-old female patient with a severity score of 5: “I feel like I talk just fine. And if people don’t understand me, they ask me again. I’ll repeat what I said.” However, in those with a higher degree of disability (n = 17 with a 5-domain NPCCSS score of 10 or greater), it was often reported that patients struggled to express their needs or were no longer able to communicate at all with those they were unfamiliar with (n = 5/17; 29.4%): “He very rarely verbally communicates. So, um I mean it’s okay for us but if [it’s] somebody from outside [the family], he can’t communicate alone.” (Caregiver of 28-year-old male with a severity score of 23).

Cognitive impairment led to difficulty at school or work for patients (n = 8; 28.6%) across all degrees of disability from 5-domain NPCCSS scores of 5 and above. However, for those at higher degrees of disability, cognitive symptoms had a greater impact on patients’ ability to communicate: “And it’s hard to, like, get her attention back on what I’m trying to convey to her.” (Caregiver of 8-year-old female with severity score of 13). Of the four (14.3%) participants who described cognitive symptoms’ impacting patients’ ability to communicate and understand, all had 5-domain NPCCSS scores of 10 or higher.

### Impact of NPC by age

Overall, impacts on patients’ ADLs were generally consistent across age with some exceptions. For instance, impacts on former activities for pediatric patients were often about no longer being able to do “child” things like playing with peers: “You know, we try to let him have fun. […] He’s not as free to do stuff as other kids would be.” (Caregiver of 8-year-old male). For adults, though, more domestic activities like writing or gardening were impacted: “I do notice when I’m out gardening or doing things like that, you know, my hand, it gets cramped.” (34-year-old male patient).

Caregivers of pediatric patients were often more concerned with their ability to keep up with peers or even attend school: “Can I send him to pre-school and he will be able to participate?” (Caregiver of 2-year-old male). For adults, though, the impact was more about being able to work or maintain work: “I work on a computer. And so many times now I need to, you know, double check what I’ve typed […] because I don’t have the fine motor skills I used to have.” (51-year-old female patient).

Daily tasks were also impacted in different ways for pediatric and adult patients. For children, caregivers discussed patients’ basic ADLs like eating and dressing: “So, dressing himself is harder, brushing his teeth is harder, you know, to where he needs assistance with it.” (Caregiver of 5-year-old male). While for adults, participants also discussed more complex tasks like managing money or finances: “He couldn’t problem solve some of the things that had come up that he previously had no issue with. Whether it be the cash register or, you know, dealing with the finances of the business.” (Caregiver of 32-year-old male).

How NPC impacted patients’ HRQOL also varied by the age of the patient. For instance, older adult patients (≥ 30 years of age) or their caregivers more commonly talked about the psychological impacts of NPC on patients (n = 5; 17.9%) than younger adult patients 18 to 29 years of age (n = 3; 10.7%) or pediatric patients under the age of 18 years (n = 2; 7.1%). These included loss in language skills: “He’s also struggling with his voice now and he can’t remember words and he’s finding it very difficult to talk fluently.” (Caregiver of 45-year-old male). They also included a change in the patient’s personality: “It impacts personality, you know, [Patient] was always a quiet person, but you know I think he’s always been a happy, outgoing person. Um- and we see that, you know, he’s not quite as much anymore because he has less interpersonal interactions.” (Caregiver of 32-year-old).

Patients’ age also impacted how caregivers’ daily activities were affected. For younger patients (29 years or younger), more caregivers expressed the need to alter their own behavior (n = 14; 50.0%) than caregivers of patients 30 years or older (n = 2; 7.1%). Conversely, more caregivers of adult patients 18 years or older talked about the patient requiring additional caregiving (n = 12; 66.7%) than caregivers of pediatric patients under the age of 18 years (n = 4; 14.3%). Relatedly, needing to guess what patients needed was particularly relevant to caregivers of pediatric patients. All caregivers who expressed this impact in the interview (n = 5; 17.9%) were caregivers of patients under the age of 18 years.

In the interviews, the unique way in which NPC impacted adult patients was a common theme. Caregivers of adults often raised issues of consent and decision making that are more difficult with adult patients than with children: “With young children you make the decisions for your children. With adults that doesn’t happen all the time and you have to make the decisions with them and that’s often difficult.” (Caregiver of 32-year-old male).

Some caregivers also mentioned how patients have more support while they are school age; however, after they finish school there is less support for them in terms of work: “I think also it’s hard coming out of education and there’s kind of nothing. It’s really hard to know what to do with a kid who’s not getting any job opportunities because there’s some difficulties, but still is too able too capable to, to just put into a, care situation, where, you know, they, they wouldn’t be engaged sufficiently.” (Caregiver of 18-year-old female).

### Impact of NPC by age of symptom onset

The impact of NPC also did not vary considerably according to the age of the patient at symptom onset. When comparing patients whose symptoms started before the age of 15 (n = 20) to those whose symptoms began after this age (n = 8), the most commonly reported impacts on the patients’ ADLs in both groups included it being more difficult to do tasks (n = 18/20; 90.0% versus n = 8/8; 100%, respectively), and having more difficulty or having to stop former activities (n = 14/20; 70.0% versus n = 5/8; 62.5%, respectively). In those whose symptoms started before the age of 15, having difficulty communicating or understanding (n = 13/20; 65.0%) and having greater dependence on caregivers (n = 11/20; 55.0%) were also commonly reported. For those whose symptoms began after the age of 15 years, impact upon school or work was also commonly reported (n = 5/8; 62.5%).

Although the general impacts felt were consistent across groups defined by age of symptom onset, precisely how or why patients had more difficulty or had to stop former activities varied. For those whose symptoms began as children, seizures or difficulties with ambulation often impacted their former activities such as playing: “There are a lot of things she used to be able to do […] she used to at least toss the ball or try to push it.” (Caregiver of 17-year-old female whose symptoms began at 10 years). For those whose symptoms began as adolescents or young adults, it was often fine motor skills having an impact on handwriting that were noted: “She was doing um, an exam she had to either use a computer or somebody that’s doing the typing for her […] because her handwriting is so slow and it’s quite uneven.” (Caregiver of 18-year-old female whose symptoms began at 13 years).

## Discussion

Symptoms relating to ambulation, swallowing, speech, fine motor skills, and cognition were rated as most important by participants, and this rating was consistent across age groups. Difficulties with ambulation and swallowing were highly salient symptoms to the participants interviewed in this study. Results also suggested that at higher levels of NPC severity, swallowing seemed to be more important than symptoms associated with ambulation or cognition. This may be because once patients reach a stage of severity in which they are not moving about independently they have less risk of falls, whereas swallowing and the life-threatening risks associated with that (e.g., choking) become the key symptom to manage. This finding of key symptoms, which were common across age groups, age of onset, and by degree of disability, is consistent with previous NPC research studies which also found these were the most important symptoms [15, 24]. It therefore suggests that these five symptoms are key for understanding the impact of NPC and should be integral to the clinical management of NPC and the evaluation of therapeutic interventions for NPC patients. It also provides additional support that the 5-domain NPCCSS measures five of the most important symptoms of NPC to caregivers and patients [25].

The impacts described by participants centered around mobility, self-care, ability to do previous activities, and emotional impacts such as feelings of anxiety or depression. Mobility issues both in terms of moving around inside and outside the house as well as issues around transportation were often reported by participants. Activities of self-care were often discussed by participants as patients experienced difficulty with personal hygiene activities, often requiring additional caregiving support for these activities. A key impact of NPC reported by participants was patients having difficulty or no longer being able to undertake previous activities and, as a result, experiencing greater social isolation. Furthermore, the emotional impacts of NPC, including feelings of frustration, anxiety, fear, and worry were often reported.

As the majority of interview participants were caregivers, the emotional impacts of NPC on patients, such as feelings of frustration, were informed by their interpretation of patients’ behavior. For example, as the caregiver of a 16-year-old male said, “People have trouble understanding him, so it’s frustrating for them, and it’s frustrating for us. Um, I think it’s frustrating for him, but he doesn’t say it’s frustrating for him.” Future research may want to confirm with patients themselves, where possible, that they experience feelings of frustration or similar impacts that are difficult for an observer to know directly.

The degree of disease disability according to the 5-domain NPCCSS scores not only reflected the severity of the patient’s NPC but also how the disease impacted daily life. For instance, at lower levels of disability, patients may need to be more mindful when moving around indoors, but they do not necessarily need to make adjustments to their homes. At higher levels of disability, though, patients may need to adjust their home environment to accommodate them. Similarly, at higher levels of disability, activities outside the home and communicating with others were more difficult, possibly leading to greater social isolation. These findings suggest that as the degree of disability progresses, the degree of impact NPC has on patients and caregivers similarly increases.

Participants in this survey and follow-up interview study were very engaged and willing to share their experiences, leading to a considerable amount of rich, qualitative data to inform understanding of the patient and caregiver experience of NPC. Findings from this study on the burden of NPC highlight areas that are most salient to patients and caregivers and which should be considered by relevant stakeholders in public health and those who support the community of NPC patients and their caregivers. The impacts NPC can have on mobility and ADLs, both inside and outside the home, and practical challenges such as transportation are of critical importance. This research has also clearly demonstrated that NPC has a dramatic impact upon both the patients and also their caregivers, in terms of ADLs and all aspects of HRQOL (social, emotional, and physical), and that support is needed to help individuals in these areas in addition to finding treatments for the symptoms of NPC.

There are some limitations of the current study and implications for future research that are important to note. In this study, the study activities were primarily completed by caregivers rather than patients. Given NPC’s impact on fine motor skills, cognition, and speech, many patients were not able to complete the web survey on their own or be interviewed over the telephone. Ethical considerations also limited participants to be either adult patients or caregivers, meaning pediatric patients could not take part in study activities on their own and their experiences of NPC symptoms and impacts could only be captured by proxy report of the caregiver. If greater self-report could be obtained, this would help further confirm that the impacts of NPC raised by caregivers in this study are also what is of most importance to patients themselves. As this study was able to include several patients self-reporting for themselves, whose responses were similar to caregivers, it provides some provisional support that the impacts reported by caregivers are also the impacts of greatest importance to patients themselves.

The study originally intended telephone interviews to be approximately 60 min; however, after the initial interviews overran, the interview time was extended to approximately 90 min. Although this may have increased participant burden, those involved were highly engaged and willing to take part in longer interviews. Given the long diagnosis process, the complexity of presentation, and the multitude of ways in which NPC impacts people’s day-to-day lives, even the extended interview time did not enable all aspects of patients’ and caregivers’ experiences to be covered. Future studies should anticipate longer interviews or may want to consider having multiple interviews with patients and caregivers to get a fuller picture of their experiences with NPC and its impacts.

## Conclusions

NPC profoundly impacts patients’ ADLs and HRQOL in a number of ways. The results confirm that ambulation, swallowing, speech, fine motor skills, and cognition are the most important manifestations. ADLs and HRQOL were impaired in the majority of patients as well as their caregivers, which was independent of current age and age of onset of symptoms. Impacts were also reported across all levels of NPC disease-related disability, although how exactly NPC impacted patients and caregivers varied by the degree of disability, with greater impact at higher levels of disease disability.

Knowing the impacts and burden of NPC on patients and caregivers is important for understanding patients’ and caregivers’ lived experience of NPC as well as for revealing the areas of support patients and caregivers need most. This study has begun to increase that understanding, pointing to the needs patients—particularly adult patients—and caregivers have in terms of greater support for providing care, transportation of the patient, more resources for adults with NPC, and emotional and mental health support for both patients and caregivers.

## Methods

### Study design

Adult (≥ 18 years of age) NPC patients, and caregivers of pediatric (< 18 years of age) or adult patients with NPC, were recruited into the study. The study had two parts: Part 1, a web-based survey; and Part 2, a follow-up telephone interview. A subset of the web-based survey participants took part in the follow-up telephone interview. To ensure the study activities (i.e., survey and interview) would be as applicable and sensitive as possible to the lived experience of patients with NPC and their caregivers, two patient advocacy groups (PAGs) provided feedback on the study design, protocol, and associated documents. Both PAGs also invited a caregiver who was a member of their organization to review the content of the web-based survey and interview and provide feedback to improve the clarity of the content, instructions, and questions.

In the web-based survey, participants (patients or caregivers) were asked to assess the patient’s NPC disease status, rating the severity of the nine major domains within the NPCCSS [25, 26]. Each item was adapted from the clinician-reported NPCCSS to be suitable for reporting by caregiver or patient while maintaining the scoring structure. Ratings of severity across these nine domains were used to calculate the patient’s 5-domain NPCCSS score (a total score is yielded ranging from 0 to 25). The 5-domain NPCCSS scores were used to stratify patients for the interview.

Participants were then asked in the survey to identify and rank the five most important NPC symptoms to them from 1 = “the very most important symptom” to 5 = “the least important symptom.” In this ranking exercise, participants could choose from 9 symptoms listed (see Table 8), which corresponded with the 9 major domains of the NPCCSS and any additional symptoms found relevant by the participant.

**Table 8:** NPC symptoms participants asked to rank in survey

NPCCSS Domain	Symptom Asked to Rank in Survey
Ambulation	Unsteadiness or clumsiness when walking about from place to place (ataxia)
Fine Motor	Difficulty with coordinating hand movements (dystonia)
Speech	Slurred or irregular speech (dysarthria)
Swallow	Difficulty swallowing (dysphagia)
Eye Movement	Difficulty moving one’s eyes up and down (vertical supranuclear gaze palsy)
Seizures	Seizures
Memory	Memory problems
Hearing	Hearing difficulties
Cognition	Learning difficulties or problems with cognitive abilities like following instructions, making decisions, or focusing attention (cognitive dysfunction or dementia)
Other	Participant could write up to three other symptoms to rank as most important

For each of the five most important symptoms ranked by participants, in addition to any of the symptoms within the 5-domain NPCCSS that were not selected, participants were asked follow-up questions to indicate the impact of that symptom on the ADLs and HRQOL for patients and caregivers respectively. Responses to these questions were given on a 5-point Likert scale of “Extremely,” “A lot,” “Moderately,” “Somewhat,” or “Not at all.”

The sub-group of participants who then went on to take part in the semi-structured telephone interviews were asked in greater detail about the five symptoms that they indicated as being important in the survey, why participants had included these symptoms in their top five, the patient’s experience of that symptom, and the impact it has had on the ADLs and HRQOL of both patients and caregivers. The interviewer also asked about the patient’s experiences with and impacts of any symptoms of the 5-domain NPCCSS that were not included in the participants ranking of the top five symptoms in the survey.

### Participant recruitment

Participant recruitment was conducted through PAGs who work closely with the NPC community. In the US, this was the National Niemann-Pick Disease Foundation (NNPDF); in the UK, it was the Niemann-Pick UK (NPUK). Each PAG advertised the research study to their member community via email blast, a post on the PAG’s website or social media profiles, or personalized emails sent to individual members. Individuals interested in participating then directly contacted the researchers to be granted access to the web-based survey. The survey included electronic informed consent. The aim of the study was to receive 60 completed surveys across the two countries. As part of the survey, participants could express interest in being contacted to take part in the follow-up telephone interview. Those who expressed such interest were contacted on a first come, first served basis to schedule the telephone interview with the aim to collect information from at least half of the survey respondents (i.e., 30 completed interviews).

## Data Availability

The datasets generated and/or analyzed during the current study are not publicly available but are available from the corresponding author on reasonable request.

## Abbreviations

ADLs: Activities of daily living
FDA: Food and Drug Administration
HRQOL: Health-related quality of life
NPC: Niemann-Pick disease type C
NPCCSS: Niemann-Pick Type C Clinical Severity Scale
PAG: Patient advocacy group
PFDD: Patient focused drug development
QOL: Quality of life
UK: United Kingdom
US: United States

## References

[1] Geberhiwot T, Moro A, Dardis A (2018). Consensus clinical management guidelines for Niemann-Pick Disease Type C. Orphanet J Rare Dis..

[2] Hammond N, Munkacsi A, Sturley S (2019). The complexity of a monogenic neurodegenerative disease: more than two decades of therapeutic driven research into Niemann-Pick type C disease. Biochim Biophy Acta Mol Cell Biol Lipids..

[3] Vanier M. Niemann-Pick disease type C. Orphanet J Rare Dis. 2010;5(16). 10.1186/1750-1172-1185-1116. 10.1186/1750-1172-5-16 PMC2902432 20525256

[4] Naureckiene S, Sleat D, Lackland H (2000). Identification of HE1 as the second gene of Niemann-Pick C disease. Science..

[5] Lloyd-Evans E, Platt F (2010). Lipids on trial: the search for the offending metabolite in Niemann-Pick Type C Disease. Traffic..

[6] Platt F, d’Azzo A, Davidson B, Neufeld E, Tifft C (2018). Lysosomal storage diseases. Nat Rev Dis Primers..

[7] Vanier M (2010). Niemann-Pick disease type C. Orphanet J Rare Dis..

[8] Wraith J, Sedel F, Pineda M (2014). Niemann-Pick type C Suspicion Index tool: analyses by association of manifestations. J Inherit Metab Dis..

[9] Patterson M, Clayton P, Gissen P (2017). Recommendations for the detection and diagnosis of Niemann-Pick disease type C: an update. Neurol Clin Prac..

[10] Stampfer M, Theiss S, Amraoui Y, Jiang X, Keller S, Ory D (2013). Niemann-Pick disease type C clinical database: cognitive and coordination deficits are early disease indicators. Orphanet J Rare Dis..

[11] Maarup TJ, Chen AH, Porter FD (2015). Intrathecal 2-hydroxypropyl-beta-cyclodextrin in a single patient with Niemann-Pick C1. Molecular genetics and metabolism..

[12] Matencio A, Navarro-Orcajada S, González-Ramón A, García-Carmona F, López-Nicolás JM. Recent advances in the treatment of niemann pick disease type c: a mini-review. Int J Pharm. 2020:119440. 10.1016/j.ijpharm.2020.119440 32428546

[13] Patterson MC, Vecchio D, Prady H, Abel L, Wraith JE (2007). Miglustat for treatment of Niemann-Pick C disease: a randomised controlled study. The Lancet Neurology..

[14] Patterson M, Mengel E, Bembi B (2020). Efficacy and safety of arimoclomol in patients with Niemann-Pick disease type C: Results from a double-blind, randomized placebo-controlled trial with a novel treatment. Molecular Genetics and Metabolism..

[15] A Parseghian. Niemann-Pick type C patient and caregiver voices: externally-led, patient-focused drug development meeting (2019). https://nnpdf.org/files/2019/09/NPC-PFDD-Voice-of-the-Patient-report-09162019.pdf. Accessed June 8, 2020.

[16] Patterson MC, Hendriksz CJ, Walterfang M, Sedel F, Vanier MT, Wijburg F (2012). Recommendations for the diagnosis and management of Niemann–Pick disease type C: An update. Molecular Genetics and Metabolism..

[17] Wraith J, Imrie J (2007). Understanding Niemann-Pick disease type C and its potential treatment.

[18] Di Lazzaro V, Marano M, Florio L, De Santis S (2016). Niemann–Pick type C: focus on the adolescent/adult onset form. International Journal of Neuroscience..

[19] Sévin M, Lesca G, Baumann N (2006). The adult form of Niemann–Pick disease type C. Brain..

[20] Walterfang M, Velakoulis D (2010). Niemann-Pick disease type C in adulthood–a psychiatric and neurological disorder. Eur Psychiatr Rev..

[21] Hessel F. Burden of disease. Encyclopedia of Public Health. Available at: https://link.springer.com/referenceworkentry/10.1007%2F978-1-4020-5614-7_297#:~:text=The%20term%20burden%20of%20disease,aspects%2C%20and%20costs%20to%20society. Accessed September 24, 2020.

[22] Edemekong PF, Bomgaars DL, Sukumaran S, et al. Activities of Daily Living (ADLs) [Updated 2020 Jun 26]. In: StatPearls [Internet]. Treasure Island (FL): StatPearls Publishing; 2020 Jan-. Available from: https://www.ncbi.nlm.nih.gov/books/NBK470404/. Accessed August 20, 2020.

[23] Office of Disease Prevention and Health Promotion. Health-Related Qualit of Life and Well-being. Available at: https://www.healthypeople.gov/2020/about/foundation-health-measures/Health-Related-Quality-of-Life-and-Well-Being#:~:text=Health%2Drelated%20quality%20of%20life%20(HRQoL)%20is%20a%20multi,has%20on%20quality%20of%20life. Accessed August 20, 2020.

[24] Cortina-Borja M, te Vruchte D, Mengel E (2018). Annual severity increment score as a tool for stratifying patients with Niemann-Pick disease type C and for recruitment to clinical trials. Orphanet journal of rare diseases..

[25] Patterson M, Lloyd-Price L, Guldberg C, et al. Validation of a short-form 5-domain Niemann-Pick type C clinical severity scale. Orphanet J Rare Dis. (in preparation). 10.1186/s13023-021-01719-2 PMC7881637 33579322

[26] Yanjanin N, Velez J, Gropman A (2010). Linear clinical progression, independent of age of onset, in Niemann-Pick Disease, Type C. Am J Med Genet B Neuropsychiatr Genet..

